# Supplementary material for: Modeling glioblastoma heterogeneity as a dynamic network of cell states
Source: Mol Syst Biol. 2021 Sep 16;17(9):e10105. doi: 10.15252/msb.202010105 (PMC8444284; doi:10.15252/msb.202010105)
Supplement: Supplementary file 6 — Source Data for Figure 5 [file MSB-17-e10105-s004.zip › Figure5A_sourcedata/GSEA_3017/nosigdb_stateF.GseaPreranked.1622795015644/RICHARDS_NOWAKOWSKI_SCIENCE_2017_MGE_IPC1_UPREG.html]

Details for gene set RICHARDS\_NOWAKOWSKI\_SCIENCE\_2017\_MGE\_IPC1\_UPREG[GSEA]

|  || Dataset | state33017 |
| Phenotype | NoPhenotypeAvailable |
| Upregulated in class | na\_neg |
| GeneSet | RICHARDS\_NOWAKOWSKI\_SCIENCE\_2017\_MGE\_IPC1\_UPREG |
| Enrichment Score (ES) | -0.68997246 |
| Normalized Enrichment Score (NES) | -5.3866754 |
| Nominal p-value | 0.0 |
| FDR q-value | 0.0 |
| FWER p-Value | 0.0 |
Table: GSEA Results Summary

  

Fig 1: Enrichment plot: RICHARDS\_NOWAKOWSKI\_SCIENCE\_2017\_MGE\_IPC1\_UPREG      
 Profile of the Running ES Score & Positions of GeneSet Members on the Rank Ordered List

  

| PROBE | GENE SYMBOL | GENE\_TITLE | RANK IN GENE LIST | RANK METRIC SCORE | RUNNING ES | CORE ENRICHMENT || 1 | RPS27L |  |  | 33 | 1.025 | -0.0116 | No |
| 2 | PON2 |  |  | 93 | 0.764 | -0.0378 | No |
| 3 | B2M |  |  | 110 | 0.736 | -0.0421 | No |
| 4 | TOB1 |  |  | 161 | 0.646 | -0.0644 | No |
| 5 | KAT2B |  |  | 193 | 0.594 | -0.0772 | No |
| 6 | PIK3R3 |  |  | 251 | 0.553 | -0.1035 | No |
| 7 | CCNG1 |  |  | 292 | 0.515 | -0.1213 | No |
| 8 | SEMA5A |  |  | 336 | 0.494 | -0.1407 | No |
| 9 | PDCD4 |  |  | 360 | 0.484 | -0.1500 | No |
| 10 | ABAT |  |  | 362 | 0.483 | -0.1480 | No |
| 11 | HSPA5 |  |  | 373 | 0.480 | -0.1506 | No |
| 12 | METTL7A |  |  | 403 | 0.469 | -0.1630 | No |
| 13 | SLC3A2 |  |  | 411 | 0.466 | -0.1642 | No |
| 14 | HIGD2A |  |  | 414 | 0.464 | -0.1628 | No |
| 15 | IKBIP |  |  | 567 | 0.408 | -0.2385 | No |
| 16 | SORT1 |  |  | 577 | 0.406 | -0.2410 | No |
| 17 | HDAC9 |  |  | 579 | 0.404 | -0.2394 | No |
| 18 | TMSB4X |  |  | 590 | 0.401 | -0.2424 | No |
| 19 | FKBP2 |  |  | 622 | 0.391 | -0.2563 | No |
| 20 | FNIP1 |  |  | 640 | 0.387 | -0.2629 | No |
| 21 | ADD3 |  |  | 674 | 0.378 | -0.2779 | No |
| 22 | BCHE |  |  | 677 | 0.376 | -0.2769 | No |
| 23 | IFIT1 |  |  | 720 | 0.365 | -0.2965 | No |
| 24 | PAIP2 |  |  | 739 | 0.362 | -0.3039 | No |
| 25 | MSI2 |  |  | 780 | 0.352 | -0.3225 | No |
| 26 | RAB3IP |  |  | 797 | 0.348 | -0.3289 | No |
| 27 | KDM4A |  |  | 861 | 0.333 | -0.3595 | No |
| 28 | ARL4A |  |  | 889 | 0.328 | -0.3716 | No |
| 29 | MDH1 |  |  | 924 | 0.322 | -0.3873 | No |
| 30 | FAT1 |  |  | 968 | 0.315 | -0.4077 | No |
| 31 | RGS3 |  |  | 969 | 0.315 | -0.4060 | No |
| 32 | AGL |  |  | 976 | 0.314 | -0.4075 | No |
| 33 | SMIM14 |  |  | 985 | 0.312 | -0.4100 | No |
| 34 | HNRNPH2 |  |  | 989 | 0.311 | -0.4099 | No |
| 35 | SRGAP2B |  |  | 1006 | 0.309 | -0.4165 | No |
| 36 | SNX3 |  |  | 1150 | 0.285 | -0.4882 | No |
| 37 | ZBTB20 |  |  | 1181 | 0.281 | -0.5021 | No |
| 38 | CKB |  |  | 1200 | 0.279 | -0.5099 | No |
| 39 | JAM2 |  |  | 1204 | 0.279 | -0.5100 | No |
| 40 | PTPRE |  |  | 1243 | 0.275 | -0.5280 | No |
| 41 | COX6C |  |  | 1252 | 0.274 | -0.5307 | No |
| 42 | GAPDH |  |  | 1361 | 0.261 | -0.5846 | No |
| 43 | KLHL9 |  |  | 1371 | 0.260 | -0.5879 | No |
| 44 | DNA2 |  |  | 1479 | -0.252 | -0.6414 | No |
| 45 | SNRPG |  |  | 1486 | -0.254 | -0.6431 | No |
| 46 | C2orf69 |  |  | 1500 | -0.259 | -0.6485 | No |
| 47 | ITGB1BP1 |  |  | 1501 | -0.259 | -0.6471 | No |
| 48 | HNRNPA2B1 |  |  | 1503 | -0.261 | -0.6463 | No |
| 49 | BRD8 |  |  | 1508 | -0.263 | -0.6469 | No |
| 50 | NIF3L1 |  |  | 1519 | -0.265 | -0.6507 | No |
| 51 | NPM1 |  |  | 1543 | -0.270 | -0.6610 | No |
| 52 | ZNF43 |  |  | 1544 | -0.270 | -0.6596 | No |
| 53 | NUP62 |  |  | 1562 | -0.275 | -0.6669 | No |
| 54 | KHDRBS1 |  |  | 1573 | -0.278 | -0.6706 | No |
| 55 | MRPL51 |  |  | 1576 | -0.279 | -0.6701 | No |
| 56 | FUBP1 |  |  | 1578 | -0.280 | -0.6692 | No |
| 57 | IQGAP3 |  |  | 1584 | -0.282 | -0.6703 | No |
| 58 | COX17 |  |  | 1589 | -0.284 | -0.6708 | No |
| 59 | ZFP36L1 |  |  | 1590 | -0.284 | -0.6694 | No |
| 60 | CDKN2C |  |  | 1593 | -0.285 | -0.6689 | No |
| 61 | RUVBL2 |  |  | 1599 | -0.286 | -0.6700 | No |
| 62 | RCC1 |  |  | 1604 | -0.286 | -0.6705 | No |
| 63 | CCDC150 |  |  | 1605 | -0.286 | -0.6690 | No |
| 64 | CNTRL |  |  | 1610 | -0.288 | -0.6696 | No |
| 65 | HP1BP3 |  |  | 1618 | -0.290 | -0.6717 | No |
| 66 | FAM83D |  |  | 1624 | -0.292 | -0.6727 | No |
| 67 | RUVBL1 |  |  | 1625 | -0.292 | -0.6712 | No |
| 68 | HES6 |  |  | 1632 | -0.294 | -0.6727 | No |
| 69 | RSRC1 |  |  | 1633 | -0.294 | -0.6712 | No |
| 70 | GAS2L3 |  |  | 1640 | -0.297 | -0.6727 | No |
| 71 | RPL8 |  |  | 1647 | -0.299 | -0.6742 | No |
| 72 | APOLD1 |  |  | 1648 | -0.299 | -0.6726 | No |
| 73 | NDUFAF3 |  |  | 1656 | -0.304 | -0.6746 | No |
| 74 | NOM1 |  |  | 1664 | -0.305 | -0.6766 | No |
| 75 | ANAPC11 |  |  | 1667 | -0.306 | -0.6761 | No |
| 76 | LSM14A |  |  | 1668 | -0.306 | -0.6745 | No |
| 77 | THOC7 |  |  | 1670 | -0.306 | -0.6734 | No |
| 78 | RDX |  |  | 1678 | -0.310 | -0.6753 | No |
| 79 | FADS1 |  |  | 1682 | -0.312 | -0.6753 | No |
| 80 | NUP107 |  |  | 1683 | -0.313 | -0.6736 | No |
| 81 | TMEM256 |  |  | 1685 | -0.314 | -0.6725 | No |
| 82 | G2E3 |  |  | 1687 | -0.315 | -0.6714 | No |
| 83 | DCXR |  |  | 1689 | -0.315 | -0.6702 | No |
| 84 | NUDCD2 |  |  | 1691 | -0.316 | -0.6691 | No |
| 85 | TNFAIP8L1 |  |  | 1695 | -0.317 | -0.6690 | No |
| 86 | MGME1 |  |  | 1697 | -0.317 | -0.6678 | No |
| 87 | CDC25C |  |  | 1724 | -0.327 | -0.6794 | No |
| 88 | VEZF1 |  |  | 1732 | -0.331 | -0.6813 | No |
| 89 | NEIL3 |  |  | 1733 | -0.331 | -0.6796 | No |
| 90 | PLK4 |  |  | 1740 | -0.336 | -0.6809 | No |
| 91 | STIL |  |  | 1744 | -0.337 | -0.6807 | No |
| 92 | EXOSC9 |  |  | 1746 | -0.338 | -0.6794 | No |
| 93 | TOP1 |  |  | 1749 | -0.340 | -0.6787 | No |
| 94 | CNIH4 |  |  | 1751 | -0.340 | -0.6774 | No |
| 95 | RAB13 |  |  | 1761 | -0.345 | -0.6802 | No |
| 96 | PPM1G |  |  | 1771 | -0.348 | -0.6830 | No |
| 97 | RAN |  |  | 1772 | -0.350 | -0.6812 | No |
| 98 | RBM8A |  |  | 1777 | -0.351 | -0.6814 | No |
| 99 | KIF20A |  |  | 1792 | -0.358 | -0.6867 | No |
| 100 | ILF2 |  |  | 1795 | -0.360 | -0.6858 | No |
| 101 | LGALS1 |  |  | 1799 | -0.362 | -0.6855 | No |
| 102 | CENPL |  |  | 1805 | -0.362 | -0.6862 | No |
| 103 | NES |  |  | 1811 | -0.370 | -0.6868 | No |
| 104 | NEK2 |  |  | 1815 | -0.371 | -0.6864 | No |
| 105 | NUP50 |  |  | 1823 | -0.376 | -0.6880 | Yes |
| 106 | RHNO1 |  |  | 1824 | -0.377 | -0.6860 | Yes |
| 107 | SAP30 |  |  | 1826 | -0.378 | -0.6846 | Yes |
| 108 | C18orf54 |  |  | 1830 | -0.380 | -0.6841 | Yes |
| 109 | LDHA |  |  | 1833 | -0.380 | -0.6832 | Yes |
| 110 | BARD1 |  |  | 1839 | -0.384 | -0.6837 | Yes |
| 111 | CCT6A |  |  | 1848 | -0.387 | -0.6858 | Yes |
| 112 | SRSF10 |  |  | 1851 | -0.390 | -0.6848 | Yes |
| 113 | CEP135 |  |  | 1855 | -0.393 | -0.6843 | Yes |
| 114 | ACTL6A |  |  | 1859 | -0.393 | -0.6838 | Yes |
| 115 | KIF18B |  |  | 1860 | -0.393 | -0.6817 | Yes |
| 116 | EXOSC8 |  |  | 1865 | -0.397 | -0.6817 | Yes |
| 117 | GDI2 |  |  | 1866 | -0.397 | -0.6796 | Yes |
| 118 | CHD7 |  |  | 1874 | -0.400 | -0.6811 | Yes |
| 119 | SYNCRIP |  |  | 1877 | -0.403 | -0.6800 | Yes |
| 120 | CENPO |  |  | 1884 | -0.410 | -0.6810 | Yes |
| 121 | RBMX |  |  | 1885 | -0.412 | -0.6788 | Yes |
| 122 | SHCBP1 |  |  | 1886 | -0.412 | -0.6767 | Yes |
| 123 | POLQ |  |  | 1891 | -0.417 | -0.6765 | Yes |
| 124 | YBX1 |  |  | 1892 | -0.417 | -0.6744 | Yes |
| 125 | SAE1 |  |  | 1893 | -0.417 | -0.6722 | Yes |
| 126 | RPL12 |  |  | 1895 | -0.418 | -0.6705 | Yes |
| 127 | DKC1 |  |  | 1904 | -0.428 | -0.6724 | Yes |
| 128 | MXD3 |  |  | 1905 | -0.428 | -0.6702 | Yes |
| 129 | ANP32A |  |  | 1912 | -0.430 | -0.6710 | Yes |
| 130 | MNS1 |  |  | 1913 | -0.430 | -0.6687 | Yes |
| 131 | DCP2 |  |  | 1914 | -0.432 | -0.6665 | Yes |
| 132 | UHRF1 |  |  | 1921 | -0.438 | -0.6673 | Yes |
| 133 | DIAPH3 |  |  | 1924 | -0.439 | -0.6660 | Yes |
| 134 | MZT1 |  |  | 1936 | -0.447 | -0.6693 | Yes |
| 135 | MZT2B |  |  | 1941 | -0.449 | -0.6690 | Yes |
| 136 | TTF2 |  |  | 1942 | -0.449 | -0.6667 | Yes |
| 137 | TPRKB |  |  | 1944 | -0.450 | -0.6648 | Yes |
| 138 | PSMA4 |  |  | 1946 | -0.451 | -0.6630 | Yes |
| 139 | CCNF |  |  | 1947 | -0.451 | -0.6606 | Yes |
| 140 | CLIC1 |  |  | 1949 | -0.454 | -0.6588 | Yes |
| 141 | DDX39A |  |  | 1957 | -0.458 | -0.6600 | Yes |
| 142 | SEPHS1 |  |  | 1967 | -0.464 | -0.6622 | Yes |
| 143 | TIMM10 |  |  | 1975 | -0.470 | -0.6633 | Yes |
| 144 | CIT |  |  | 1978 | -0.474 | -0.6618 | Yes |
| 145 | SKA1 |  |  | 1996 | -0.490 | -0.6680 | Yes |
| 146 | SPAG5 |  |  | 1997 | -0.490 | -0.6654 | Yes |
| 147 | TRA2B |  |  | 1998 | -0.490 | -0.6629 | Yes |
| 148 | ARL6IP1 |  |  | 2001 | -0.494 | -0.6613 | Yes |
| 149 | FAM111A |  |  | 2002 | -0.497 | -0.6587 | Yes |
| 150 | LRR1 |  |  | 2004 | -0.497 | -0.6566 | Yes |
| 151 | TUBB6 |  |  | 2006 | -0.500 | -0.6545 | Yes |
| 152 | XPO1 |  |  | 2007 | -0.500 | -0.6519 | Yes |
| 153 | HNRNPUL1 |  |  | 2009 | -0.501 | -0.6498 | Yes |
| 154 | RANGAP1 |  |  | 2011 | -0.502 | -0.6477 | Yes |
| 155 | CDK5RAP2 |  |  | 2012 | -0.503 | -0.6451 | Yes |
| 156 | CALM3 |  |  | 2022 | -0.516 | -0.6470 | Yes |
| 157 | SRRT |  |  | 2025 | -0.517 | -0.6453 | Yes |
| 158 | HAUS8 |  |  | 2026 | -0.517 | -0.6426 | Yes |
| 159 | PARPBP |  |  | 2030 | -0.520 | -0.6415 | Yes |
| 160 | DEPDC1B |  |  | 2037 | -0.527 | -0.6418 | Yes |
| 161 | CDCA3 |  |  | 2041 | -0.529 | -0.6406 | Yes |
| 162 | CCT5 |  |  | 2042 | -0.529 | -0.6378 | Yes |
| 163 | EIF5A |  |  | 2043 | -0.529 | -0.6350 | Yes |
| 164 | TROAP |  |  | 2046 | -0.533 | -0.6333 | Yes |
| 165 | TTK |  |  | 2047 | -0.533 | -0.6305 | Yes |
| 166 | KIF22 |  |  | 2053 | -0.541 | -0.6302 | Yes |
| 167 | CENPJ |  |  | 2054 | -0.544 | -0.6274 | Yes |
| 168 | SAPCD2 |  |  | 2058 | -0.545 | -0.6261 | Yes |
| 169 | INCENP |  |  | 2061 | -0.556 | -0.6242 | Yes |
| 170 | SPDL1 |  |  | 2064 | -0.569 | -0.6222 | Yes |
| 171 | PTMA |  |  | 2065 | -0.570 | -0.6193 | Yes |
| 172 | FANCD2 |  |  | 2068 | -0.577 | -0.6173 | Yes |
| 173 | ZWINT |  |  | 2069 | -0.577 | -0.6143 | Yes |
| 174 | CEP152 |  |  | 2070 | -0.578 | -0.6113 | Yes |
| 175 | RTKN2 |  |  | 2071 | -0.579 | -0.6082 | Yes |
| 176 | RACGAP1 |  |  | 2072 | -0.579 | -0.6052 | Yes |
| 177 | CENPA |  |  | 2073 | -0.586 | -0.6021 | Yes |
| 178 | MND1 |  |  | 2074 | -0.586 | -0.5991 | Yes |
| 179 | SKA3 |  |  | 2077 | -0.590 | -0.5970 | Yes |
| 180 | BUB3 |  |  | 2078 | -0.596 | -0.5939 | Yes |
| 181 | AURKB |  |  | 2079 | -0.598 | -0.5908 | Yes |
| 182 | SRSF3 |  |  | 2081 | -0.606 | -0.5881 | Yes |
| 183 | CENPK |  |  | 2082 | -0.607 | -0.5850 | Yes |
| 184 | HNRNPR |  |  | 2083 | -0.612 | -0.5818 | Yes |
| 185 | USP1 |  |  | 2084 | -0.612 | -0.5786 | Yes |
| 186 | KNSTRN |  |  | 2085 | -0.615 | -0.5754 | Yes |
| 187 | CKAP5 |  |  | 2086 | -0.623 | -0.5721 | Yes |
| 188 | PLK1 |  |  | 2087 | -0.624 | -0.5689 | Yes |
| 189 | PIF1 |  |  | 2088 | -0.627 | -0.5656 | Yes |
| 190 | NCAPD2 |  |  | 2089 | -0.627 | -0.5623 | Yes |
| 191 | KIF18A |  |  | 2093 | -0.636 | -0.5605 | Yes |
| 192 | MIS18A |  |  | 2096 | -0.639 | -0.5582 | Yes |
| 193 | BRCA2 |  |  | 2100 | -0.650 | -0.5564 | Yes |
| 194 | PHF19 |  |  | 2101 | -0.650 | -0.5530 | Yes |
| 195 | ITGB3BP |  |  | 2102 | -0.652 | -0.5496 | Yes |
| 196 | NCAPH |  |  | 2104 | -0.652 | -0.5467 | Yes |
| 197 | CKAP2L |  |  | 2109 | -0.663 | -0.5453 | Yes |
| 198 | AURKA |  |  | 2110 | -0.673 | -0.5417 | Yes |
| 199 | CKS2 |  |  | 2115 | -0.694 | -0.5402 | Yes |
| 200 | CDC20 |  |  | 2117 | -0.695 | -0.5371 | Yes |
| 201 | RAD21 |  |  | 2118 | -0.697 | -0.5334 | Yes |
| 202 | ANP32B |  |  | 2119 | -0.700 | -0.5298 | Yes |
| 203 | CDCA2 |  |  | 2120 | -0.705 | -0.5261 | Yes |
| 204 | HDGF |  |  | 2124 | -0.709 | -0.5239 | Yes |
| 205 | RAD51AP1 |  |  | 2127 | -0.715 | -0.5212 | Yes |
| 206 | CDCA8 |  |  | 2129 | -0.722 | -0.5180 | Yes |
| 207 | CKAP2 |  |  | 2131 | -0.729 | -0.5147 | Yes |
| 208 | VRK1 |  |  | 2134 | -0.743 | -0.5118 | Yes |
| 209 | TUBA1C |  |  | 2135 | -0.756 | -0.5079 | Yes |
| 210 | KIF2C |  |  | 2136 | -0.756 | -0.5039 | Yes |
| 211 | KIF5B |  |  | 2137 | -0.756 | -0.5000 | Yes |
| 212 | KIF14 |  |  | 2141 | -0.773 | -0.4975 | Yes |
| 213 | NDC80 |  |  | 2142 | -0.775 | -0.4934 | Yes |
| 214 | H2AFV |  |  | 2143 | -0.779 | -0.4893 | Yes |
| 215 | FBXO5 |  |  | 2144 | -0.779 | -0.4853 | Yes |
| 216 | KIF23 |  |  | 2145 | -0.782 | -0.4812 | Yes |
| 217 | GMNN |  |  | 2146 | -0.785 | -0.4771 | Yes |
| 218 | LMNB2 |  |  | 2147 | -0.786 | -0.4730 | Yes |
| 219 | MELK |  |  | 2148 | -0.786 | -0.4689 | Yes |
| 220 | ANLN |  |  | 2149 | -0.797 | -0.4647 | Yes |
| 221 | SMC3 |  |  | 2150 | -0.800 | -0.4606 | Yes |
| 222 | KIFC1 |  |  | 2151 | -0.802 | -0.4564 | Yes |
| 223 | DEPDC1 |  |  | 2152 | -0.809 | -0.4521 | Yes |
| 224 | DEK |  |  | 2154 | -0.817 | -0.4484 | Yes |
| 225 | NCAPG2 |  |  | 2156 | -0.827 | -0.4446 | Yes |
| 226 | TUBB |  |  | 2157 | -0.831 | -0.4402 | Yes |
| 227 | NUCKS1 |  |  | 2158 | -0.832 | -0.4359 | Yes |
| 228 | LSM5 |  |  | 2159 | -0.835 | -0.4315 | Yes |
| 229 | TUBB4B |  |  | 2162 | -0.843 | -0.4282 | Yes |
| 230 | ECT2 |  |  | 2163 | -0.846 | -0.4238 | Yes |
| 231 | HMGB3 |  |  | 2164 | -0.852 | -0.4193 | Yes |
| 232 | KIF20B |  |  | 2165 | -0.852 | -0.4149 | Yes |
| 233 | SPC24 |  |  | 2166 | -0.853 | -0.4104 | Yes |
| 234 | ARHGAP11A |  |  | 2167 | -0.855 | -0.4059 | Yes |
| 235 | DTYMK |  |  | 2168 | -0.858 | -0.4015 | Yes |
| 236 | BUB1 |  |  | 2170 | -0.860 | -0.3975 | Yes |
| 237 | FOXM1 |  |  | 2171 | -0.861 | -0.3930 | Yes |
| 238 | CDCA5 |  |  | 2172 | -0.866 | -0.3885 | Yes |
| 239 | LBR |  |  | 2173 | -0.870 | -0.3839 | Yes |
| 240 | BUB1B |  |  | 2174 | -0.871 | -0.3794 | Yes |
| 241 | NUF2 |  |  | 2176 | -0.877 | -0.3753 | Yes |
| 242 | CEP55 |  |  | 2177 | -0.883 | -0.3707 | Yes |
| 243 | KPNA2 |  |  | 2178 | -0.894 | -0.3660 | Yes |
| 244 | HMMR |  |  | 2179 | -0.895 | -0.3614 | Yes |
| 245 | CCDC34 |  |  | 2180 | -0.898 | -0.3567 | Yes |
| 246 | CCNA2 |  |  | 2181 | -0.902 | -0.3520 | Yes |
| 247 | MIS18BP1 |  |  | 2183 | -0.903 | -0.3478 | Yes |
| 248 | KIF15 |  |  | 2185 | -0.915 | -0.3435 | Yes |
| 249 | KIF4A |  |  | 2186 | -0.921 | -0.3387 | Yes |
| 250 | PRR11 |  |  | 2187 | -0.930 | -0.3338 | Yes |
| 251 | ORC6 |  |  | 2188 | -0.937 | -0.3289 | Yes |
| 252 | C21orf58 |  |  | 2189 | -0.937 | -0.3241 | Yes |
| 253 | CENPW |  |  | 2192 | -0.943 | -0.3202 | Yes |
| 254 | TUBA1B |  |  | 2193 | -0.947 | -0.3152 | Yes |
| 255 | LMNB1 |  |  | 2194 | -0.950 | -0.3103 | Yes |
| 256 | HJURP |  |  | 2195 | -0.962 | -0.3052 | Yes |
| 257 | UBE2T |  |  | 2196 | -0.965 | -0.3002 | Yes |
| 258 | CDKN3 |  |  | 2199 | -0.979 | -0.2961 | Yes |
| 259 | CCNB2 |  |  | 2200 | -0.983 | -0.2910 | Yes |
| 260 | CKS1B |  |  | 2201 | -0.987 | -0.2858 | Yes |
| 261 | HIST1H1E |  |  | 2202 | -0.992 | -0.2806 | Yes |
| 262 | ESCO2 |  |  | 2204 | -1.004 | -0.2759 | Yes |
| 263 | SMC2 |  |  | 2205 | -1.006 | -0.2707 | Yes |
| 264 | KIF11 |  |  | 2206 | -1.008 | -0.2654 | Yes |
| 265 | EZH2 |  |  | 2207 | -1.017 | -0.2601 | Yes |
| 266 | SPC25 |  |  | 2208 | -1.019 | -0.2548 | Yes |
| 267 | CENPE |  |  | 2211 | -1.036 | -0.2504 | Yes |
| 268 | CENPN |  |  | 2217 | -1.079 | -0.2473 | Yes |
| 269 | UBE2C |  |  | 2218 | -1.081 | -0.2417 | Yes |
| 270 | CENPU |  |  | 2219 | -1.088 | -0.2360 | Yes |
| 271 | H2AFZ |  |  | 2220 | -1.090 | -0.2303 | Yes |
| 272 | HMGB1 |  |  | 2221 | -1.116 | -0.2245 | Yes |
| 273 | NCAPG |  |  | 2222 | -1.122 | -0.2186 | Yes |
| 274 | H2AFX |  |  | 2223 | -1.146 | -0.2126 | Yes |
| 275 | CCNB1 |  |  | 2224 | -1.148 | -0.2066 | Yes |
| 276 | CDK1 |  |  | 2225 | -1.153 | -0.2006 | Yes |
| 277 | PTTG1 |  |  | 2226 | -1.198 | -0.1943 | Yes |
| 278 | ANP32E |  |  | 2227 | -1.200 | -0.1881 | Yes |
| 279 | MAD2L1 |  |  | 2228 | -1.204 | -0.1818 | Yes |
| 280 | UBE2S |  |  | 2229 | -1.205 | -0.1755 | Yes |
| 281 | GTSE1 |  |  | 2230 | -1.220 | -0.1691 | Yes |
| 282 | DBF4 |  |  | 2232 | -1.247 | -0.1631 | Yes |
| 283 | HMGN2 |  |  | 2233 | -1.267 | -0.1565 | Yes |
| 284 | RRM2 |  |  | 2234 | -1.268 | -0.1499 | Yes |
| 285 | DLGAP5 |  |  | 2235 | -1.273 | -0.1432 | Yes |
| 286 | SMC4 |  |  | 2236 | -1.281 | -0.1366 | Yes |
| 287 | PBK |  |  | 2237 | -1.325 | -0.1296 | Yes |
| 288 | ATAD2 |  |  | 2238 | -1.381 | -0.1224 | Yes |
| 289 | TMPO |  |  | 2239 | -1.382 | -0.1152 | Yes |
| 290 | PRC1 |  |  | 2240 | -1.397 | -0.1079 | Yes |
| 291 | HMGB2 |  |  | 2241 | -1.469 | -0.1002 | Yes |
| 292 | BIRC5 |  |  | 2242 | -1.469 | -0.0926 | Yes |
| 293 | HIST1H1C |  |  | 2243 | -1.485 | -0.0848 | Yes |
| 294 | ASPM |  |  | 2244 | -1.490 | -0.0770 | Yes |
| 295 | TOP2A |  |  | 2246 | -1.562 | -0.0694 | Yes |
| 296 | NUSAP1 |  |  | 2247 | -1.600 | -0.0610 | Yes |
| 297 | TPX2 |  |  | 2248 | -1.609 | -0.0526 | Yes |
| 298 | CENPF |  |  | 2249 | -1.691 | -0.0438 | Yes |
| 299 | HIST1H1D |  |  | 2250 | -1.720 | -0.0348 | Yes |
| 300 | HIST1H1B |  |  | 2251 | -1.796 | -0.0255 | Yes |
| 301 | MKI67 |  |  | 2252 | -1.892 | -0.0156 | Yes |
| 302 | HIST1H4C |  |  | 2253 | -2.984 | 0.0000 | Yes |
Table: GSEA details [plain text format]

  

Fig 2: RICHARDS\_NOWAKOWSKI\_SCIENCE\_2017\_MGE\_IPC1\_UPREG: Random ES distribution      
 Gene set null distribution of ES for **RICHARDS\_NOWAKOWSKI\_SCIENCE\_2017\_MGE\_IPC1\_UPREG**
